# Structured physical exercise improves neuropsychiatric symptoms in acute dementia care: a hospital-based RCT

**DOI:** 10.1186/s13195-017-0289-z

**Published:** 2017-08-29

**Authors:** Tim Fleiner, Hannah Dauth, Marleen Gersie, Wiebren Zijlstra, Peter Haussermann

**Affiliations:** 1 Institute of Movement and Sport Gerontology, German Sport University Cologne, Am Sportpark Muengersdorf 6, 50933 Köln, Germany; 2 LVR-Hospital Cologne, Department of Geriatric Psychiatry & Psychotherapy, Academic Teaching Hospital of the University of Cologne, Wilhelm-Griesinger-Straße 23, 51109 Köln, Germany

**Keywords:** Dementia, Hospital, Neuropsychiatric signs and symptoms, Exercise, Physical activity, Social stimulation, Antipsychotic medication, Sedative medication

## Abstract

**Background:**

The primary objective of this trial is to investigate the effects of a short-term exercise program on neuropsychiatric signs and symptoms in acute hospital dementia care.

**Methods:**

Within a hospital-based randomized controlled trial, the intervention group conducted a 2-week exercise program with four 20-min exercise sessions on 3 days per week. The control group conducted a social stimulation program. Effects on neuropsychiatric signs and symptoms were measured via the Alzheimer’s Disease Cooperative Study-Clinical Global Impression of Change, the Neuropsychiatric Inventory, and the Cohen-Mansfield Agitation Inventory. The antipsychotic and sedative dosage was quantified by olanzapine and diazepam equivalents.

**Results:**

Eighty-five patients were randomized via minimization to an intervention group (IG) and a control group (CG). Seventy patients (82%) (mean age 80 years, 33 females, mean Mini Mental State Examination score 18.3 points) completed the trial. As compared to the CG (*n* = 35), the IG (*n* = 35) showed significantly reduced neuropsychiatric signs and symptoms. Especially, agitated behavior and lability improved. There were no between-group differences concerning antipsychotic and benzodiazepine medication.

**Conclusions:**

This exercise program is easily applicable in hospital dementia care and significantly reduces neuropsychiatric signs and symptoms in patients suffering from predominantly moderate stages of dementia.

**Trial registration:**

German Clinical Trial Register DRKS00006740. Registered 28 October 2014.

## Background

Neuropsychiatric signs and symptoms in dementia cover a broad range of symptoms with depression, agitation, and apathy being most common. They affect almost every patient in the course of the disease [1]. These behavioral and psychological symptoms in dementia seriously impact caregiver burden and often lead to admission to geriatric or geriatric psychiatry hospital wards or to specialized dementia care units in nursing homes.

Treatment of neuropsychiatric signs and symptoms is a key challenge in acute dementia care. As the treatment of neuropsychiatric signs and symptoms with antipsychotic medication may lead to harmful side-effects [2], strategies to reduce the use of antipsychotic medication are urgently warranted [3]. Structured physical activation is increasingly being considered as a worthwhile nonpharmacological treatment approach for neuropsychiatric signs and symptoms. Scherder et al. [4] postulated a direct link between physical inactivity and increased agitation in patients with dementia. First trials in long-term dementia care units confirm this hypothesis, showing reduced agitated behavior and affective symptoms through participation in exercise programs [5–10]. These first results should be interpreted with caution because they are based on conceptual reviews and observational studies-randomized controlled exercise trials investigating the effects on neuropsychiatric signs and symptoms are mostly lacking [11, 12]. Taken together, there is a lack of evidence for physical activation as a treatment approach in the acute hospital dementia care setting.

### Objective and hypothesis

The primary objective of this trial is to investigate the effects of a short-term exercise program on neuropsychiatric signs and symptoms in acute hospital dementia care. Effects on symptom dimensions as well as the use of psychotropic medication were analyzed as secondary outcome measures.

#### Study hypothesis

The intervention group, carrying out a short-term exercise program in addition to treatment as usual (TAU), is hypothesized to exhibit reduced neuropsychiatric signs and symptoms at follow-up as compared to the control group, receiving a social stimulation program in addition to TAU.

## Methods

### Study design

An RCT was conducted in the LVR-Hospital Cologne on three specialized dementia care wards in the Department of Geriatric Psychiatry. Patients were randomly allocated to an intervention group (IG), carrying out a 2-week exercise program, and a control group (CG), conducting a 2-week social stimulation program. Pre and post assessment was conducted 3 days before and after the intervention respectively. For further details, refer to the study protocol published previously [13]. The RCT has been registered in the German Clinical Trial Register (DRKS00006740) and has been approved by the local ethics committee.

### Patients

All patients were assessed for their eligibility by two senior geriatric psychiatrists, who were not part of the study team. The following inclusion criteria were applied: diagnosis of dementia according to ICD-10 [14]; a minimum length of stay of 1 week before enrollment into the study in order to help patients become familiarized with the ward setting and to exclude delirium; clinical exclusion of delirium based on the validated German version of the Confusion Assessment Method [15, 16]; ability to perform the Timed Up and Go Test [17]; and written informed consent from the patient’s legal guardian as well as from the patient, if possible. According to the sample size calculation published in the study protocol [13], each group should include 53 patients respectively. Therefore, an enrollment of 130 patients was planned. An external scientist allocated patients randomly to the IG or to the CG (1:1 allocation ratio) via minimization [18].

### Outcomes

The investigation of the trial’s primary objective, the overall effects of the intervention on neuropsychiatric signs and symptoms, was based on the Alzheimer’s Disease Cooperative Study-Clinical Global Impression of Change (ADCS-CGIC) [19, 20]. This proxy-based interview was conducted with the patient’s primary nurse, who rated the change in the patient’s neuropsychiatric signs and symptoms at follow-up compared to the baseline measurement. This 7-point rating ranges from ‘very much improved’ (1 point) to ‘no change’ (4 points) to ‘very much worse’ (7 points). The following dimensions were rated: emotional agitation (emotional distress and affective symptoms), lability, psychomotor agitation, verbal aggression, and physical aggression.

Effects on different dimensions of neuropsychiatric signs and symptoms have been rated using the Neuropsychiatric Inventory (NPI) [21] and the Cohen-Mansfield Agitation Inventory (CMAI) [22]. Both the NPI and the CMAI were conducted at baseline and follow-up measurement by rating the patients’ neuropsychiatric signs and symptoms over 1 week retrospectively. These proxy-based interview ratings were carried out by experienced and blinded investigators, who were responsible for interviewing both the caregiver as well as the medical and nursing staff. The individual dosage of sedative and antipsychotic medication was recorded at baseline, during the intervention, and at follow-up. Antipsychotic medication was converted to the olanzapine equivalent dosage (OED) and the benzodiazepine dosage to the diazepam equivalent dosage (DED) [23, 24].

### Interventions

The IG conducted an ‘exercise carrousel program’ for 2 weeks [13]. On 3 days per week, four 20-min exercise sessions per day were conducted. Within the day-structuring exercise schedule, strengthening exercises with ankle or wrist-worn weights or endurance exercises for lower and upper limbs on seated ergometers were carried out in groups of three patients. Exercise protocols with instructions, repetitions, intensity, individually tailoring of the exercises, and recording of the exercise adherence have been reported in the study protocol [13].

Within the 2-week intervention period, the CG conducted a social stimulation program of attended table games (120 min/week), instructed by the hospital’s occupational therapists.

### Statistical methods

Statistical analyses were carried out with SPSS (IBM SPSS Statistics version 23.0). Sample characteristics are reported as mean (SD) for continuous variables and number (%) for categorical variables. Differences between group characteristics (Table 1) were analyzed via χ^2^ test for categorical variables, Mann–Whitney *U* test for ordinal variables, and unpaired *t* test for continuous variables.

**Table 1:** Patient characteristics

	Intervention group (*n* = 35)					Control group (*n* = 35)					
	*n*	Mean	SD	Min	Max	*n*	Mean	SD	Min	Max	*p*
Age		80	7	67	91		80	7	68	92	0.50
Female (%)	16 (46)					17 (49)					0.81
Body mass index (kg/m^2^)	35	25.4	4	19	32	35	25.6	3.9	18	32	0.99
ICD-10 dementia diagnosis											
Alzheimer's disease (F00), *n* (%)	8 (23)					18 (51)					0.05
Vascular dementia (F01), *n* (%)	6 (17)					3 (9)					0.28
Mixed type (F02 + F03), *n* (%)	19 (54)					13 (37)					0.15
Dementia in Parkinson's disease (F02.3), *n* (%)	2 (6)					0					0.15
Lewy-body dementia (G31.8), *n* (%)	0					1 (3)					0.31
Mini Mental Status Examination (points/30)	35	18.4	4.8	7	26	35	18.3	4.7	8	26	0.92
Demtect (points/18)	27	5.2	3.2	0	14	25	5	3	1	10	0.48
Clock Drawing Test (points/6)	33	4.2	1.6	1	6	30	4.7	1.7	1	6	0.09
Cognitive reserve (years of education)	35	12	1.7	8	18	34	13	3.5	7	18	0.93
Bayer Activities of Daily Living (points/10)	35	8.2	1	4.4	9.5	35	7.8	1.3	4.9	9.4	0.41
Timed Up and Go test (s)	35	13.7	4.9	7.3	27	35	13.1	2.8	8.2	18.8	0.31
10 Meter Gait Speed (m/s)	35	0.8	0.2	0.4	1.3	35	0.8	0.2	0.3	1.4	0.40

Patients’ characteristics are presented as mean, standard deviation (*SD*), minimum (*Min*) and maximum (*Max*) for continuous variables and number (%) for categorical variables. Statistical differences (*p*) between the groups were calculated by χ^2^ test for nominal data and *t* test for continuous variables. *ICD-10* classification of mental and behavioural disorders

The effects on neuropsychiatric signs and symptoms were analyzed by unpaired *t* tests (ADCS-CGIC) as group differences at follow-up measurement. The trial’s secondary objectives, the effects on single dimensions of neuropsychiatric signs and symptoms (NPI and CMAI), were analyzed by a two-way repeated-measurement analysis of variances. Possible effects of time, group, and time × group interaction were analyzed with (un)paired *t* tests as post-hoc tests. Then, in a next step, the effects of the patients’ dementia diagnoses (Alzheimer’s disease vs non-Alzheimer’s disease) were analyzed as a covariate.

The dosage of neuroleptic and sedative medication was analyzed by one-factor analysis of variances with paired or unpaired *t* test as post-hoc tests if the results were normally distributed. If the results were not normally distributed, a Friedman analysis of variances was performed for within-group analysis with the Wilcoxon rank test as a post-hoc test. Between-group differences were analyzed using the Mann–Whitney *U* test. Effect sizes were calculated as Cohen’s *d* [25], *d* < 0.2 indicating no effect, *d* = 0.2–0.4 indicating a small effect, *d* = 0.5–0.7 indicating a moderate effect, and *d* > 0.8 indicating a large effect.

An intention-to-treat analysis was applied for all analyses. Within the two-sided testing, the level of significance was set at *p* ≤ 0.05. Post-hoc test levels of significance were adapted by Bonferroni correction.

## Results

### Patient flow and sample characteristics

**Fig. 1:**
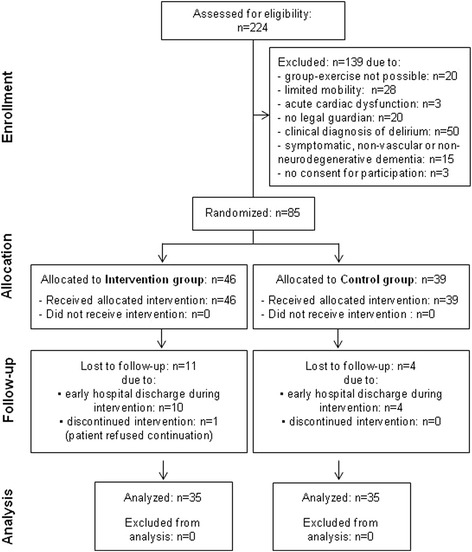
Study flow chart

From December 1, 2014, to December 31, 2015, a total of 224 patients suffering from dementia were screened for eligibility into the trial (Fig. 1): 62% (*n* = 139) of these patients were not eligible for inclusion, mostly due to clinical diagnosis of delirium (*n* = 50; 22%). Out of the 85 patients randomized to the IG and the CG, 15 (18%) patients were lost to follow-up, most of them due to early hospital discharge. Eighty-two percent of the allocated patients finished the intervention period, completed the follow-up measurement, and were included in the final analysis.

The group characteristics of the patients who completed the study protocol (*N* = 70) are presented in Table 1. Mean age was 80 (SD = 6) years, 33 (47%) women completed the trial, and the mean MMSE score of the whole sample was 18.3 (SD = 4.8) points. In summary, both the IG as well as the CG can be characterized as a patient population with predominantly moderate dementia, moderate overall neuropsychiatric signs and symptoms, and especially an overall low level of psychotic symptoms (Table 2). There were no significant differences in the clinical characteristics of the IG and the CG except for more patients suffering from Alzheimer’s disease in the CG (*p* = 0.05).

**Table 2:** Effects on neuropsychiatric signs and symptoms

	Intervention group, mean (SD)		Control group, mean (SD)		RM ANOVA group × time			
	Baseline	Follow-up	Baseline	Follow-up	*F*(1,68)	*p*	*d*	1 – *β*
NPI total^a^	22.5 (12.3)	10.3 (7.3)	22.5 (13.9)	16.2 (9.9)	4.4	0.04	0.51	0.99
Psychotic symptoms	3.3 (5.9)	1.0 (1.9)	2.8 (4.6)	1.9 (3.3)	1.6	0.22	0.30	0.70
Emotional symptoms	11.0 (7.3)	4.0 (4.2)	10.7 (9.0)	7.2 (5.9)	3.7	0.06	0.75	0.99
Behavior symptoms	6.9 (4.9)	4.0 (4.4)	6.1 (4.5)	5.2 (4.6)	4.6	0.04	0.52	0.99
Neurovegetative symptoms	1.3 (2.7)	1.3 (2.8)	2.9 (4.6)	1.9 (3.1)	0.8	0.39	0.21	0.41
CMAI total^b^	51.4 (12.5)	41.7 (10.2)	51.3 (12.4)	45.5 (10.7)	2.6	0.11	0.40	0.90
Aggressive behavior	15.9 (5.1)	12.9 (1.8)	16.5 (5.8)	14.1 (2.9)	<0.1	0.57	0.42	0.21
Physically nonaggressive behavior	16.3 (7.4)	13.9 (7.5)	16.8 (6.1)	14.9 (6.7)	0.2	0.70	0.11	0.15
Verbally agitated behavior	11.3 (5.9)	7.4 (4.1)	9.7 (4.4)	8.8 (4.4)	7.9	0.01	0.68	0.99
Hiding and hoarding	2.3 (1.5)	2.4 (1.5)	2.7 (2.3)	2.3 (1.1)	2.3	0.33	0.24	0.50

Number of patients: *n* = 35 in each group

*CMAI* Cohen-Mansfield Agitation Inventory, *NPI* Neuropsychiatric Inventory, d effect size (Cohen’s *d*), F *F*-ratio from ANOVA (between-group degrees of freedom, within-group degrees of freedom), p statistical significance, *RM-ANOVA* repeated-measures analysis of variance, *1 –* β test power, *SD* standard deviation

^a^NPI range and scaling, 0–144 points (0 meaning no symptoms); dimensions: psychotic symptoms, 0–24 points (0 meaning no symptoms); emotional symptoms, 0–48 points (0 meaning no symptoms); behavior symptoms, 0–48 points (0 meaning no symptoms); neurovegetative symptoms, 0–24 points (0 meaning no symptoms)

^b^CMAI range and scaling, 29–203 points (29 meaning no symptoms); dimensions: aggressive behavior, 12–84 points (12 meaning no symptoms); physically nonaggressive behavior, 6–42 points (6 meaning no symptoms); verbally agitated behavior, 4–28 points (4 meaning no symptoms); hiding and hoarding, 2–14 points (2 meaning no symptoms)

### Adherence to the protocol and adverse events

On average, the IG completed 128 min per week in the exercise program (SD = 53 min; minimum = 30 min/week; maximum = 200 min/week). Of these 35 patients, 18 patients (51%) participated ≥ 150 min/week, 10 patients (29%) participated 60–149 min/week, and 7 patients (20%) participated < 60 min/week. Within the exercise group, *n* = 10 patients were included with advanced stages of dementia (MMSE score ≤ 15). These patients participated in mean on 129 min/week (SD = 51 min; minimum = 50 min/week; maximum = 200/week). This participation is not different from patients with MMSE score > 15 (*n* = 25; mean = 127 min/week; SD = 54 min; minimum = 30 min/week; maximum = 200 min/week). Patients in the CG participated 105 min/week (SD = 26 min, minimum = 30 min/week; maximum = 120 min/week). There was a significantly higher adherence rate in the IG as compared to the CG (*z* = –2.55, *p* = 0.01, *d* = –0.64, 1 – *β* = 0.99).

No serious adverse events (SAE) occurred in the IG, while there were two SAE in the CG. One patient suffered from severe hyponatremia and another patient suffered from cardiac decompensation. Both SAE led to a short-term intensive care unit treatment. According to the expert opinion of an independent senior geriatric psychiatrist, there was no relation between the SAE and the study protocol.

### Effects on neuropsychiatric signs and symptoms

**Fig. 2:**
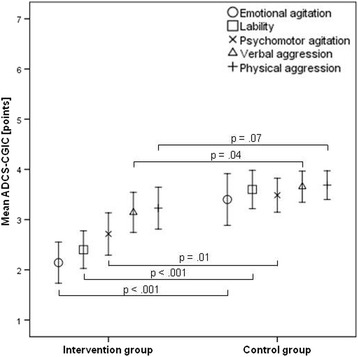
Effects on neuropsychiatric signs and symptoms (ADCS-CGIC). *ADCS-CGIC* Alzheimer’s Disease Cooperative Study-Clinical Global Impression of Change (range 1–7 points: 1 = very much improved; 4 = no change; 7 = very much worse)

The effects on neuropsychiatric signs and symptoms, as measured by the ADCS-CGIC, indicated a general decrease of neuropsychiatric signs and symptoms over the two intervention weeks in both the IG as well as the CG (Fig. 2). Compared to the CG, the IG showed significantly more positive clinical effects on the ADCS-CGIC dimensions ‘emotional agitation’ (*t*(68) = –3.89, *p* < 0.001, *d* = –0.9, 1 – *β* = 0.96), ‘lability’ (*t*(68) = –4.55, *p* < 0.001, *d* = –1.1, 1 – *β* = 0.99), ‘psychomotor agitation’ (*t*(68) = –2.91, *p* = 0.01, *d* = –0.7, 1 – *β* = 0.82), and ‘verbal aggression’ (*t*(68) = –2.06, *p* = 0.04, *d* = –0.5, 1 – *β* = 0.54). No significant differences were found in the ADCS-CGIC-category ‘physical aggression’ (*t*(68) = –1.84, *p* = 0.07, *d* = –0.4, 1– *β* = 0.38) between the groups.

The analyses on single dimensions of neuropsychiatric signs and symptoms (Table 2) showed significant reductions from baseline to follow-up for both the NPI total score (IG Δ = –12 points; CG Δ = –6 points) and the CMAI total score (IG Δ = –10 points; CG Δ = –6 points). According to Zuidema et al. [26], a change of 11 points within the NPI total score and a change of 8 points within the CMAI total score is considered to be clinically relevant.

**Fig. 3:**
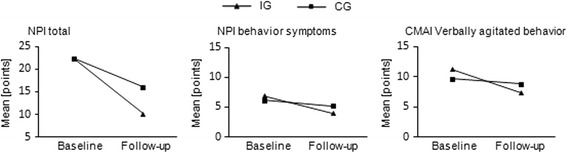
Effects on neuropsychiatric sign and symptoms (NPI and CMAI): significant group × time interactions. NPI range and scaling, 0–144 points (0 meaning no symptoms); dimension ‘behavior symptoms’, 0–48 points (0 meaning no symptoms). CMAI range and scaling, 29–203 points (29 meaning no symptoms); dimension ‘verbally agitated behavior’, 4–28 points (4 meaning no symptoms). *IG* intervention group, *CG* control group, *NPI* Neuropsychiatric Inventory; *CMAI* Cohen-Mansfield Agitation Inventory

The RM-ANOVA revealed significant group × time interactions for the total NPI, the NPI dimension ‘behavioral symptoms’, and the CMAI subscore ‘verbally agitated behavior’ (see Fig. 3). The post-hoc tests (Bonferroni-adjusted *p* = 0.0125) showed a significant drop for the NPI total score within the IG from baseline to follow-up (*t*(34) = –4.8, *p* = < 0.001) and for the CG from baseline to follow-up (*t*(34) = 2.8, *p* = 0.009). There was no significant difference between the groups at baseline, but a significantly lower NPI total score in the IG at follow-up (*t*(68) = –2.9, *p* = 0.006). Post-hoc tests for the NPI subscale ‘behavioral symptoms’ showed a significant drop within IG from baseline to follow-up (*t*(34) = 4.8, *p* < 0.001) but not for the CG. The post-hoc analysis of the CMAI subscore ‘verbally agitated behavior’ showed a significant drop within the IG from baseline to follow-up in the IG (*t*(34) = 4.6, *p* < 0.001), but not in the CG. Controlling for the patients’ dementia diagnosis (Alzheimer’s disease *n* = 26, non-Alzheimer’s disease *n* = 44) revealed no influence on the neuropsychiatric signs and symptoms as measured by the NPI total score (*F*(1,66) = 0.29, *p* = 0.60) and the CMAI total score (*F*(1,66) = 2.29, *p* = 0.14).

### Use of antipsychotic and sedative medication

There were no significant differences between the dosage of antipsychotic and sedative medication between both groups at baseline, during the intervention period, or at follow-up measurement (Table 3).

**Table 3:** Use of neuroleptic and sedative medication

	Intervention group					Control group								
Medication	*n*	Mean	SD	Min	Max	*n*	Mean	SD	Min	Max	*z*	*p*	*d*	1 – *β*
OED T0 (mg)	30	2.51	2.48	0.27	12.69	32	2.27	2.07	0.16	8.85	–0.59	0.55	–0.15	0.09
OED INT (mg)	32	2.84	3.00	0.06	12.69	30	2.66	2.05	0.09	6.87	–0.31	0.76	–0.08	0.06
OED T1 (mg)	31	2.77	3.28	0.27	12.7	29	3.05	2.06	0.21	6.69	–1.69	0.09	–0.45	0.39
DED T0 (mg)	9	3.62	1.38	1.67	5.83	11	3.65	3.72	0.11	12.5	–0.80	0.42	–0.36	0.12
DED INT (mg)	14	2.76	2.72	0.18	8.75	13	1.80	1.77	0.18	5.8	–0.76	0.45	–0.30	0.46
DED T1 (mg)	8	5.99	5.22	0.83	16.67	4	1.64	0.85	0.83	2.81	–1.45	0.15	–0.92	0.55

*OED* olanzapine equivalent dosage, *DED* diazepam equivalent dosage, *T0* baseline, *INT* intervention period, *T1* follow-up, *SD* standard deviation, *Min* minimum, *Max* maximum, z Mann–Whitney *U* test *z*-score (samples not normally distributed), p statistical significance, d effect size (Cohen’s *d*), *1 –* β test power

The analysis of the sedative dosage (DED) over time also showed no significant differences in the IG (χ^2^ = 4.11, 2 df, *N* = 14, *p* = 0.13). In the CG, there was a significant reduction of sedative medication over time (χ^2^ = 11.31, 2 df, *N* = 13, *p* = 0.004). Here, Wilcoxon post-hoc analysis (Bonferroni correction: *p* ≤ 0.016) indicated a significant reduction in the sedative dosage (*z* = –3.182, *p* = 0.001, *r* = –0.77, 1 – β = 0.99) between the intervention period (*n* = 13 patients with a mean dosage of 1.80 mg/day) and the follow-up measurement (*n* = 4 patients with a mean dose of 1.64 mg/day). There were no statistical significant differences within the CG from baseline to the intervention period and from baseline to follow-up measurement.

## Discussion

The primary objective of this hospital-based RCT was to investigate the effects of a short-term exercise program on exacerbated neuropsychiatric signs and symptoms. The analysis of the psychopathometric rating scales revealed a significant reduction of overall neuropsychiatric signs and symptoms for the IG as compared to the CG after a 2-week intervention period. We found clinically relevant effect sizes (*r* ≥ 0.5) with appropriate test power (1 – *β* ≥ 0.80) in four of the five ADCS-CGIC dimensions (Fig. 2). These results are further affirmed by the total NPI score, the NPI dimension ‘behavioral symptoms’, and the CMAI subscore ‘verbally agitated behavior’, which all show clinically relevant behavioral improvements in the IG, but not in the CG [26] (Table 2 and Fig. 3). The observed effects on neuropsychiatric signs and symptoms could not be explained by different use of benzodiazepine or neuroleptic medication in either of the groups (Table 3). Concerning neuroleptic medication, we found no significant differences between the IG and the CG before, during, or after the intervention period. Nearly all patients received antipsychotic medication. One-third of the patients in both groups were on benzodiazepine medication at baseline, and only eight patients in the IG and three patients in the CG were on benzodiazepine medication at follow-up measurement (Table 3). In the IG, there was a nonsignificant increase of benzodiazepine dosage. In the CG, there was a slight but significant decrease from 1.80 to 1.64 mg/day, which we do not consider to be clinically relevant. We saw a drop in the number of patients in the CG who received sedative medication between baseline and follow-up measurement from 11 to 4. Given comparable dosages of antipsychotic and sedative medication as well as a similar level of social stimulation, this structured short-term exercise program significantly improved neuropsychiatric signs and symptoms in the IG.

Earlier intervention studies, which were mostly of longer duration, have indicated a significant reduction of affective symptoms by physical exercise, while other neuropsychiatric signs and symptoms did not improve [8, 9, 27]. In our study, we also found a significant reduction of depressive symptoms in the IG. Furthermore, we saw a significant decrease of the total neuropsychiatric scores (ADCS-CGIC, NPI) and the neuropsychiatric dimensions ‘behavior symptoms’ (NPI) and ‘verbally agitated behavior’ (CMAI) (Table 2). The analyses of covariance revealed that the dementia diagnosis group had no impact on the effects of the exercise intervention on dimensions of neuropsychiatric signs and symptoms (NPI total, CMAI total).

A pivotal trial looking in more detail at neuropsychiatric signs and symptoms and the effects of antipsychotic medication is the CATIE-AD study [28]. The CATIE-AD trial investigated the effects on neuropsychiatric signs and symptoms of a 12-week intervention with risperidone (ADCS-CGIC mean = 2.6 (±1.5)), quetiapine (ADCS-CGIC mean = 2.7 (±1.1)), olanzapine (ADCS-CGIC mean = 2.9 (±1.3)), and placebo (ADCS-CGIC mean = 3.3 (±1.5)) treatment. In our 2-week intervention study, we found very similar improvements of neuropsychiatric signs and symptoms (IG ADCS-CGIC mean = 2.7 (±0.8)) compared to the CATIE-AD risperidone and quetiapine groups and better results as compared to the CATIE-AD olanzapine group [28].

The IG participated in a mean of 128 min (SD = 53 min) of structured exercise per week. Eighteen patients (51%) of the IG were in compliance with the recommendations of the American College of Sports Medicine [29], suggesting 150 min of physical activity per week for older people to achieve health benefits. In this RCT, more than 50% of the patients were able to comply with the aforementioned recommendations for healthy older people, suggesting that these recommendations might also be useful for older demented patients. The adherence rate in this RCT was quite high as compared to other studies [6, 7]. There was no significant difference in the adherence to the exercise protocol in the group of patients suffering from advanced stages of dementia as compared to the group of patients with mild to moderate dementia. Therefore, we think this approach of applying multiple short bouts of exercise sessions during a day is well feasible for patients with advanced stages of dementia. This is an important aspect of our exercise intervention, showing similarity to multiple short-bout high-intensity interval sessions from exercise science and sports medicine [13]. Thus, a translation of exercise approaches from healthy older people to demented patients seems to be feasible. If patients are unwilling or unable to participate in an exercise session, there is the possibility to participate in the next session on the same day. This is a more flexible way of applying physical exercise as compared to routine hospital or nursing home care. Future exercise trials in dementia care will probably have to focus on ways of increasing exercise intensity and approaches to increase adherence rates in this specific patient population [7]. In this regard, our RCT provides a practicable and innovative way of providing structured physical exercise in acute dementia care.

Implementing a physical exercise program into hospital dementia care of geriatric or geriatric psychiatry wards is a crucial aspect of this RCT. This short-term trial can be especially relevant for the acute dementia care situation, as the usual length of stay ranges from 2 to 6 weeks [30]. With a period of familiarization to the new setting, pre and post measurements, and a 2-week intervention period, each patient was included in this trial for 4 weeks. Another important aspect of this trial is a well-characterized study sample, not only based on sociodemographic but also on clinical, neuroimaging, neuropsychopathometric, and geriatric assessment variables. As compared to preexisting trials, we recruited a rather large sample size and achieved a high adherence to the exercise protocol. Validated and clinically accepted neuropsychopathometric tools (ADCS-CGIC, NPI, CMAI) were applied, and not only total results on neuropsychiatric signs and symptoms but also effects on single dimensions and symptom clusters were reported. The results of this trial reveal no effects on neuropsychiatric signs and symptoms as measured by the total CMAI score. These findings are comparable to the 3-week exercise trial conducted by Aman and Thomas [6]. This may be due to a lack of sensitivity of the CMAI to detect effects of short-time exercise interventions on neuropsychiatric signs and symptoms in dementia care. Furthermore, antipsychotic and benzodiazepine drug dosages were analyzed and we controlled for the level of social stimulation. These are important methodological characteristics when investigating the effects of nonpharmacological interventions on neuropsychiatric signs and symptoms in patients suffering from dementia [7, 10].

Concerning trial limitations, several aspects need to be taken into account. Both groups had a relatively small sample size of 35 patients. However, other studies investigating the effects of exercise programs on neuropsychiatric signs and symptoms in acute dementia care had about the same [31] or markedly lower sample sizes as compared to our study [6, 27, 32]. We found these clinically relevant and highly significant effects in our relatively small sample size. Moreover, in our trial, we found a small drop-out rate of only 18% (*n* = 15) during the intervention. In the context of clinical research with patients suffering from advanced dementia, higher drop-out rates have been reported [33]. Most of the drop-outs were early discharges from hospital due to the need for rapid geriatric rehabilitation or nursing home placement. Putative blinding problems in the use of proxy-based psychopathometrics represent a further limitation of this trial. Although we have tried to assure the concealment of allocation through the whole trial (e.g., by applying exercise interventions in quiet corners of the wards), attentive nursing staff could have noticed to which study group a patient had been allocated. This may have, in some cases, influenced the psychopathometric rating. The statistical analysis included the trial completers with measurement at baseline and follow-up. Eleven patients in the IG and four patients in the CG were lost to follow-up measurement. Over both groups, 14 patients were lost due to early discharge. One patient in the IG refused to continue the exercise program. Taken together, 14/15 of the patients lost to follow-up were lost due to organizational reasons (i.e., availability of specialized nursing home places). From our view, there was no systematic reason inherent to the intervention that caused discontinuation of the study. The interventions do not have an impact on the availability of nursing home places. Therefore we do not think that a systematic error biased our results in the IG and thus we included only completers in both arms of the study. Taking these limitations into account, the following conclusions can be drawn: a 2-week exercise program with multiple short-bout exercise sessions per day is an innovative and feasible approach for structured physical activation in acute dementia care leading to clinically significant improvement of neuropsychiatric signs and symptoms. Especially, emotional agitated symptoms and lability symptoms were significantly reduced. These results may further contribute to the strongly required evidence effects of exercise on neuropsychiatric signs and symptoms. In order to be able to endorse specific physical activity programs for acute dementia care, more RCTs with structured exercise programs will have to be conducted in the future. Further investigations should also focus on neurobiological effects and underlying mechanisms of the relationship between physical activity and neuropsychiatric signs and symptoms [10, 12]. Evaluating and implementing innovative exercise approaches for patients with dementia may lead to higher adherence rates and higher levels of physical activity, thereby reducing patients’ neuropsychiatric signs and symptoms as well as the caregiver’s burden.

## Conclusions

The exercise-carrousel program is easily applicable in hospital dementia care and significantly reduces neuropsychiatric signs and symptoms in patients suffering from predominantly moderate stages of dementia.

## Abbreviations

ADCS-CGIC: Alzheimer’s Cooperative Study-Clinical Global Impression of Change
CG: Control group
CMAI: Cohen-Mansfield Agitation Inventory
DED: Diazepam equivalent dosage
IG: Intervention group
NPI: Neuropsychiatric Inventory
OED: Olanzapine equivalent dosage
RCT: Randomized controlled trial
RM-ANOVA: Repeated-measures analysis of variance
SAE: Severe adverse event
TAU: Treatment as usual
